# Neoadjuvant chemotherapy with trastuzumab, docetaxel, and carboplatin administered every 3 weeks for Japanese women with HER2-positive primary breast cancer: efficacy and safety

**DOI:** 10.1007/s10147-017-1136-8

**Published:** 2017-05-25

**Authors:** Ikuko Sugitani, Shigeto Ueda, Takashi Sakurai, Takashi Shigekawa, Eiko Hirokawa, Hiroko Shimada, Hideki Takeuchi, Kazuo Matsuura, Misono Misumi, Nobuko Fujiuchi, Takao Takahashi, Takahiro Hasebe, Akihiko Osaki, Toshiaki Saeki

**Affiliations:** 1 Department of Breast Oncology, Saitama Medical University International Medical Center, 1397-1 Yamane, Hidaka, Saitama 350-1298 Japan; 2 Department of Palliative Medicine, Saitama Medical University International Medical Center, 1397-1 Yamane, Hidaka, Saitama Japan 350-1298; 3 Department of Pathology, Saitama Medical University International Medical Center, 1397-1 Yamane, Hidaka, Saitama 350-1298 Japan; 4 Breast Center, JCHO Saitama Medical Center, 4-9-3, Kita-urawa, Urawa-ku, Saitama, 330-0074 Japan; 5 Department of Breast Surgery, Hiroshima Prefectural Hospital, 1-5-54 Kanda, Minami-ku, Hiroshkloima-Shi, Hiroshima 734-8530 Japan

**Keywords:** Breast cancer, Neoadjuvant chemotherapy, Carboplatin, Docetaxel, Trastuzumab

## Abstract

**Background:**

This phase II neoadjuvant study evaluated the efficacy and safety of a triweekly regimen of docetaxel and carboplatin in combination with trastuzumab (TCbH) in Japanese women with human epidermal growth factor receptor type2 (HER2)-positive primary breast cancer.

**Methods:**

Patients with HER2-positive, stage I–III invasive breast cancer received six courses of trastuzumab (8 mg/kg loading dose, then 6 mg/kg, day 1), docetaxel (75 mg/m^2^, day 1), and carboplatin (area under the curve: 6, day 1) every 3 weeks. The primary endpoint was pathological complete response (pCR) of both breast and axillary lymph node disease.

**Results:**

Fifty patients were enrolled in this study. Median age was 58 (range 32–75) years. All patients underwent definitive surgery. Thirty-three (66%) patients completed the chemotherapy course, while the treatment was delayed or discontinued in the other 17 (34%) patients because of adverse events (AEs). The pCR rate was 52%; the overall response rate was 66%. Grade 3/4 AEs due to nonhematological toxicity were anorexia (4%), diarrhea (2%), and rash (2%), and those due to hematological toxicity were neutropenia (36%), anemia (12%), and thrombocytopenia (2%).

**Conclusion:**

Although the triweekly six-course regimen of TCbH achieved a high pCR rate, hematological AEs frequently occurred during the latter part of the chemotherapy course. One-third of patients experienced delayed or discontinued chemotherapy.

Clinical registration number: http://www.umin.org.auUMIN000013513.

**Electronic supplementary material:**

The online version of this article (doi:10.1007/s10147-017-1136-8) contains supplementary material, which is available to authorized users.

## Introduction

Neoadjuvant chemotherapy is widely employed for patients with locally advanced breast cancer and, sometimes, even in those with operable breast cancer for the purpose of downstaging to facilitate breast-conserving surgery. Survival outcomes of such a strategy were reported to be comparable to those of adjuvant chemotherapy [1]. Patients who achieve pathological complete response (pCR) are known to have better long-term survival than those who achieve a lesser-grade response. Especially in patients with human epidermal growth factor receptor 2 (HER2)-positive breast cancer who received chemotherapy in combination with trastuzumab, an increase in pCR rate was directly related to improved survival outcomes [2, 3].

The significant improvement in outcomes of HER2-positive breast cancer patients achieved with the synergistic use of anti-HER2 drugs with cytotoxic agents in the neoadjuvant setting has prompted the evaluation of various regimens [3–5].

A regimen including docetaxel, carboplatin, and trastuzumab (TCbH) has shown promising results in some clinical trials [6–8]. In a large randomized trial (the Breast Cancer International Research Group 006, BCIRG-6), patients with HER2-positive early breast cancer were assigned doxorubicin and cyclophosphamide followed by docetaxel every 3 weeks (AC-T), the same regimen plus trastuzumab (AC-TH), or TCbH; rates of disease-free survival at 5 years were 75, 84, and 81%, respectively, and rates of overall survival were 87, 92, and 91%, respectively [9]. The latter two regimens, which both contained trastuzumab, showed equivalent clinical results. Further, both were found to be superior to AC-T. Owing to the significantly lower rates of congestive heart failure and cardiac dysfunction observed with TCbH, it is expected to become an alternative to the AC-TH regimen.

TCbH has shown promising results in the neoadjuvant setting [10]. In a multicenter neoadjuvant study, GETN(A)-1, 70 patients with HER2-positive stage II/III operable breast cancer received docetaxel (75 mg/m^2^) and carboplatin [area under curve (AUC) of 6] every 3 weeks plus weekly trastuzumab (initial dose 4 mg/kg, followed by 2 mg/kg). The pCR rate (ypT0/isypN0) was 39%, which translated into an objective response rate (ORR) of 95%, and the breast conservation rate was 64% [11].

To the best of our knowledge, the efficacy and safety of the TCbH regimen in the neoadjuvant setting has not been evaluated among Japanese women with HER2-positive breast cancer.

We conducted a neoadjuvant phase II clinical trial of docetaxel (75 mg/m^2^) and carboplatin (AUC of 6) in combination with trastuzumab (loading dose 8 mg/kg, followed by 6 mg/kg) administered every 3 weeks to Japanese women with stage 1–3 non-inflammatory breast cancer. In this report, we present the efficacy and safety profiles from this study, based on intention-to-treat analysis of data from all 50 patients.

## Patients and methods

This prospective clinical study was conducted among treatment-naïve Japanese women (age group 18–75 years) with unilateral breast cancer (T1-3, N0-2, M0), which was confirmed to be HER2-positive invasive carcinoma on histopathological examination. Eligibility criteria included: Eastern Cooperative Oncology Group (ECOG) performance status 0 or 1; adequate bone marrow reserve (absolute neutrophil count >1500/µL; platelet count >100,000/µL), renal function (serum creatinine <1.5 × upper limit of normal), and hepatic function (total bilirubin <1.5 × upper limit of normal); left ventricular ejection fraction (LVEF) within normal limits by echocardiography.

Exclusion criteria were: patients who had bilateral or metastatic breast disease, inflammatory breast cancer; pregnant or lactating women; another histological subtype or neoplasm; history of atrial or ventricular arrhythmia, congestive heart failure, myocardial infarction; psychiatric disorders; active infection.

All patients were registered at the Saitama Medical University International Medical Center and Saitama Medical Center. All patients were required to undergo breast examination and radiological evaluation as part of tumor assessment before and after neoadjuvant chemotherapy. Baseline computed tomography and bone scan or positron emission tomography were performed to detect distant metastasis. Cardiac tests (ECG and echocardiography) were performed and cardiac function including LVEF was assessed before and after treatment. Patients were withdrawn if they developed clinical symptoms of cardiac insufficiency or showed a remarkable reduction in LVEF below 50%.

Written informed consent was obtained from all participants prior to their inclusion in the study. The study was designed and conducted in accordance with good clinical practices and the Declaration of Helsinki. The institutional review board approved the study protocol and the informed consent form (11-066-2). The study was registered with the University Hospital Medical Information Network (UMIN000013513).

### Immunohistochemistry

Immunohistochemical (IHC) examination was routinely performed at baseline to determine estrogen receptor (ER), progesterone receptor (PgR), and HER2 status [12]. If IHC-HER2 was 2+, HER2 status determined by fluorescent in situ hybridization (FISH) was required for all patients. IHC 3+ or FISH ≥2.1 was defined as HER2 positivity.

### Treatment

Neoadjuvant chemotherapy consisted of carboplatin, docetaxel, and trastuzumab administered every 21 days for six courses. A loading dose of trastuzumab was administered [8 mg/kg intravenous (IV) infusion administered over a period of 90 min], followed by a subsequent dose of trastuzumab (6 mg/kg IV infusion over 60 min, every 21 days). The chemotherapeutic regimen comprised docetaxel (75 mg/m^2^ as 60-min IV infusion every 21 days) immediately followed by carboplatin (AUC of 6 as 60-min IV infusion every 21 days) with standard recommended dexamethasone (9.9 mg) and antiemetics (dexamethasone, chlorpheniramine, cimetidine). The carboplatin dose was calculated with the Calvert formula using the glomerular filtration rate calculated according to the method developed by Cockroft and Gault [13]. In the present study protocol, serum creatinine (sCre) levels ≤0.7 mg/dL were recorded as 0.7, while sCre >0.7 mg/dL were used interchangeably in the Calvert formula. Docetaxel–carboplatin were initially administered 2 days after the first injection of an initial loading dose of trastuzumab, and were then repeated during the subsequent courses.

Blood tests were carried out at day 1 of each course, prior to its initiation. Chemotherapy was withdrawn in the event of repeated episodes of hemotological toxicity or any grade 3 nonhematological toxicity and grade 4 toxicity. A treatment delay of up to 2 weeks was allowed in the event of any toxicity; otherwise, dose reduction was considered. Use of granulocyte colony-stimulating factor (G-CSF) was allowed in the case of febrile neutropenia (FN) or a high possibility of FN. The American Society of Clinical Oncology (ASCO) guidelines for the use of G-CSF were followed [14]. Chemotherapy dose was modified by one dose level for afebrile grade 4 neutropenia >7 days, grade 4 FN, grade 4 thrombocytopenia, and grade 3/4 nonhemotological AEs. Patients experiencing these AEs had a dose level reduction for both drugs (i.e., carboplatin AUC = 5 and docetaxel 60 mg/m^2^), and patients experiencing a second occurrence of grade 3/4 AEs had a second dose level reduction (i.e., carboplatin AUC = 4 and docetaxel 45 mg/m^2^).

Chemotherapy was discontinued in patients who showed progressive disease or severe AEs. After the completion of neoadjuvant chemotherapy, breast surgery and axillary intervention was performed within 5 weeks after the last course. Patients underwent mastectomy or breast-conserving surgery with sentinel lymph node biopsy or/and axillary dissection. Adjuvant radiotherapy/endocrine therapy was administered according to the institutional practice guidelines. Adjuvant trastuzumab (loading dose 8 mg/kg, followed by 6 mg/kg every 21 days) was administered for 36 weeks.

### Tumor response and toxicity assessment

Clinical response was evaluated by two-dimensional measurement on magnetic resonance imaging (MRI) or ultrasonography (US) according to the Response Evaluation Criteria in Solid Tumors (RECIST) [15], which classifies the response into five categories. Partial response (PR) was defined as a decrease of at least 30% in tumor maximum diameters, and progressive disease (PD) was defined as an increase of at least 20% in the sum of all tumor diameters from that at baseline. Disease that was neither PR nor PD was categorized as stable disease (SD). Tumor shrinkage was evaluated using the same device (MRI or US) at baseline and prior to surgery. ORR was defined as the presence of confirmed CR or confirmed PR.

Histological response after the completion of chemotherapy was evaluated by at least two pathologists (including an experienced breast pathologist, T.H.) according to the Histopathological Criteria for Assessment of Therapeutic Response in Breast cancer (2007 version) [16], based on breast and axillary lymph node resection specimens. In this study, pCR was defined as no evidence of a residual invasive component in both the breast and axilla (grade 3, ypT0/is ypN0). The disappearance or marked degeneration of two-thirds or more of the tumor cells was defined as substantially effective (grade 2). The disappearance or marked degeneration of less than two-thirds was defined as moderately effective (grade 1). Almost no change in cancer cells after treatment was defined as not effective (grade 0). Toxicity after six courses of chemotherapy was evaluated according to the Common Terminology Criteria for Adverse Events version 5.0 (CTCAE ver.5). The criteria included symptoms such as allergic reactions and cardiovascular, skin, gastrointestinal, hematologic, renal, neurologic, and infectious conditions. The primary endpoint of the study was the pCR rate; the secondary endpoints were clinical response and toxicity profile associated with the treatment.

### Statistics

The minimum expected pCR rate after the completion of chemotherapy including docetaxel and carboplatin was estimated to be 15% based on the neoadjuvant study by Hurley et al. [17]. Therefore, doubling the rate by obtaining a pCR rate of 35% with the addition of trastuzumab was considered to be sufficient to demonstrate the rationale for this regimen. With a two-sided significance level of 0.05 and a power of 0.9, the minimum sample size was calculated to be 44. Fifty patients were enrolled into this protocol to detect evidence of treatment activity. Analyses were conducted using MedCalc^Ⓡ^ version 14.12.0 (MedCalc Software bvba, Ostend, Belgium).

## Results

Between December 2012 and July 2015, 50 patients with stage I–III HER2-positive breast cancer were enrolled. All patients received at least two courses of chemotherapy. Forty-nine patients underwent definitive surgery. One patient was excluded from the pathological analysis as she did not undergo definitive surgery. Data pertaining to all 50 patients were included in the baseline patient demographics (Table 1). Median age was 58 (range 32–75) years and the median tumor size was 40 (range 18–89) mm. Forty-five (90%) patients had invasive ductal carcinoma, 1 (2%) patient had invasive lobular carcinoma, and 2 (4%) had special types. Four (8%) patients had clinical stage T1 disease, while 33 (66%) and 13 (26%) had stage T2 and T3 disease, respectively. Twenty-five (50%) patients had hormone-receptor (HR)-positive breast cancer and others had HR-negative breast cancer. 12, 68, and 20% of the patients with HR-positive breast cancer were in stages 1, 2, and 3, respectively, whereas 4, 64, and 32% of those with HR-negative breast cancer were in stages 1, 2, and 3, respectively. There were no significant differences in age, tumor size, and nodal involvement between the two groups when stratified by ER status (see the table in the Electronic supplementrary material, ESM).

**Table 1:** Baseline patient demographics (*n* = 50)

Parameter	*n*	(%)
Age, years		
Median	58	
Range	32–75	
Menopausal status		
Premenopausal	19	(38)
Postmenopausal	31	(62)
Histology		
Invasive ductal carcinoma	45	(90)
Invasive lobular carcinoma	1	(2)
Special types	2	(4)
Invasive carcinoma	2	(4)
Clinical tumor stage		
T1	4	(8)
T2	33	(66)
T3	13	(26)
Clinical nodal stage		
N0	17	(34)
N1≦	33	(66)
ER/PgR status		
Positive	25	(50)
Negative	25	(50)
HER2 status		
0/1+/2+ (FISH−)	0	(0)
3+/2+ (FISH+)	50	(100)

*ER* estrogen receptor, *PgR* progesterone receptor, *HER2* human epidermal growth factor receptor type 2, *FISH* fluorescent in situ hybridization

Pathological complete response (ypT0/is ypN0) was achieved in 26 (53%) patients in the ITT population and in 19 (57.5%) patients in the per-protocol population (Table 2). Based on the RECIST criteria, CR and PR were 10 and 56%, respectively, which corresponded to an ORR of 66%. Twenty-nine (58%) patients underwent breast-conserving surgery. For other patients, mastectomy was performed because of a large amount of residual disease, patient preference, or patient desire for breast reconstruction.

**Table 2:** Therapeutic results

Parameter	ITT population (*n* = 50)		Per-protocol population (*n* = 33)	
	*n*	(%)	*n*	(%)
Histological response				
Grade 3 (pCR in breast and axilla)	26	(52)	19	(57.5)
Grade 3 (pCR in breast)	28	(56)	21	(63.6)
Grade 2	13	(26)	8	(24.2)
Grade 1	6	(12)	4	(12.1)
Grade 0	2	(4)	0	(0)
Not assessed	1	(2)		
Overall response rate prior to surgery				
CR	5	(10)	5	(15.1)
PR	28	(56)	25	(75.7)
SD	15	(30)	3	(9)
PD	1	(2)	0	(0)
Not assessed	1	(2)		
Definitive surgery				
Mastectomy	20	(40)	11	(33.3)
Breast-conserving surgery	29	(58)	22	(66.6)
Not done	1	(2)		

*ITT* intention to treat, *pCR* pathological complete response, *CR* complete response, *PR* partial response, *SD* stable disease, *PD* progressive disease

### Treatment exposure

Thirty-three (66%) patients completed the six courses of chemotherapy on schedule. In 17 (34%) patients, a treatment delay, dose reduction, or discontinuation of chemotherapy was necessary because of AEs. Four (8%) and seven (14%) patients received primary and secondary prophylaxis with G-CSF, respectively. All patients received adjuvant trastuzumab every 21 days after surgery. The relative dose intensity (RDI), defined as the ratio of the delivered to the planned dose, was 81.8, 78.5, and 80.8% for trastuzumab, carboplatin, and docetaxel, respectively.

### Safety

**Fig. 1:**
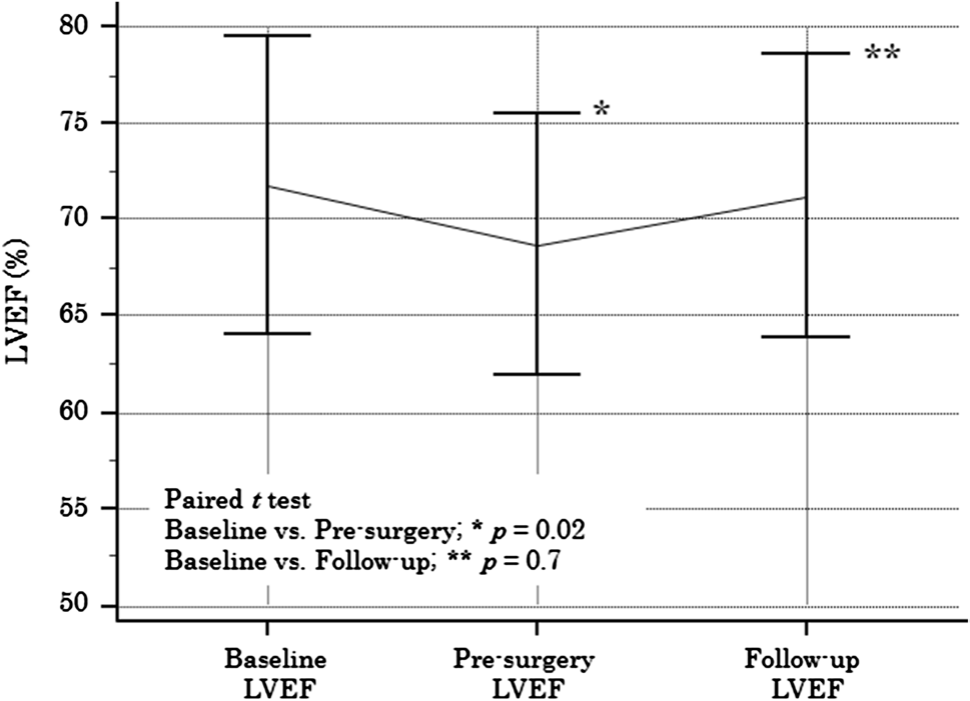
Left ventricular ejection fraction

The profiles of all grade AEs are listed in Table 3. Frequencies of nonhematological grade 3/4 AEs were: anorexia *n* = 2, 4%; diarrhea *n* = 1, 2%; and skin rash *n* = 1, 2%. The most common grade 3/4 hematological AEs were neutropenia (*n* = 18, 36%), anemia (*n* = 6, 12%), and thrombocytopenia (*n* = 1, 2%). FN occurred in 3 (6%) patients. None of the patients developed treatment-related congestive heart failure (CHF). As shown in Fig. 1, a significant decrease in mean LVEF (mean 68.7 ± 6.7 SD) after treatment was observed when compared with the baseline LVEF (mean 71.5 ± 7.7 SD, *p* = 0.02). At the completion of chemotherapy, a relative decrease in LVEF of over 20% was seen in two (4%) patients, but none of the patients experienced a decrease in LVEF of ≤50%. No change between baseline LVEF and follow-up LVEF (mean 71.2 ± 7.3 SD, *p* = 0.7) was observed when monitoring the LVEFs of patients receiving adjuvant trastuzumab 3–6 months after the surgery.

**Table 3:** Selected adverse events on six-course chemotherapy (*n* = 50)

Event	All grades		Grade 3/4	
	*n*	(%)	*n*	(%)
Nonhematologic toxicities				
Infection with neutropenia	3	(6)	3	(6)
Anaphylaxis	0	0	0	0
Mouth ulcer	22	(44)	0	0
Anorexia	42	(84)	2	(4)
Nausea	33	(66)	0	0
Vomiting	12	(24)	0	0
Diarrhea	18	(36)	1	(2)
Constipation	22	(44)	0	0
Rash	22	(44)	1	(2)
Hair loss	50	(100)	0	0
Arthralgia	14	(28)	0	0
Muscular pain	37	(74)	0	0
Peripheral neuropathy	27	(54)	0	0
Infusion reaction	8	(16)	0	0
Cardiac disorders	1	(2)	0	0
Hematologic toxicities				
Anemia	43	(86)	6	(12)
Neutropenia	26	(52)	18	(36)
Thrombocytopenia	29	(58)	1	(2)
Hepatic dysfunction	14	(28)	0	0

## Discussion

In this phase II neoadjuvant study in Japanese patients with HER2-positive breast cancer, the implementation of a TCbH regimen resulted in a high pCR rate (52%). The ORR was 66%. The pCR rate in the present study is acceptable when compared to those reported elsewhere. Mehta et al. [18] reported that patients with HER2-positive breast cancer who were assigned to a regimen of AC every 3 weeks or AC every 2 weeks followed by 13 weekly courses of paclitaxel (80 mg/m^2^), carboplatin (AUC of 2), and trastuzumab (4 mg/kg, followed by 2 mg/kg) showed pCR rates of 87.5 and 75%, respectively. Bruno et al. [11] reported a pCR of 39% with the TCbH regimen in the GENT(A)-1 study described earlier.

According to the per-protocol analysis performed in the present study, pCR rates for patients with HR-positive and HR-negative breast cancers were 53.8 and 45.8%, respectively. Although the previous meta-analysis demonstrated a trend for higher pCR rates in non-luminal/HER2 than in luminal/HER2 tumors [23], this discrepancy might be explained by the findings of a recent translational study which showed that PIK3CA mutations are associated with reduced rates of pCR to anti-HER2 therapy in HER2-positive tumors irrespective of HR status [19] [20]. Based on the evaluation of PIK3CA mutations in 504 tumor samples from patients with HER2 in the neoadjuvant studies, including GeparQuattro, GeparQuinto, and GeparSixto, the pCR rate was 11.3% for PIK3CA mutations and 27.5% for wild-type PIK3CA (OR, 0.34; 95% CI, 0.15–0.78; *p* = 0.01) among 291 patients with luminal/HER2 tumors, whereas the rate was 30.4% for PIK3CA mutations and 40.1% for wild-type PIK3CA (OR, 0.65; 95% CI, 0.32–1.32; *p* = 0.2) among 213 patients with non-luminal/HER2 tumors. Multivariable analysis revealed that the HR and PIK3CA statuses provided independent predictive information. Therefore, to improve the predictive accuracy for therapeutic response, intrinsic subtypes of HER2-positive breast cancer should be stratified by molecular biomarkers, such as PIK3CA mutations, in future clinical studies.

Most AEs were manageable. Grade 3/4 neutropenia (36%), anemia (12%), and thrombocytopenia (2%) were the most frequent AEs. Three (6%) patients experienced FN. Thrombocytopenia and anemia were of concern in the neoadjuvant setting. Sixty-six percent of the patients completed the six-course chemotherapy, while 34% of the patients required prolongation, dose reduction of chemotherapy, or they discontinued the regimen after five or fewer courses of chemotherapy because of AEs. The frequency of hematologic AEs was almost in agreement with those reported from previous trials of TCbH in the neoadjuvant/adjuvant settings [9, 11]. The use of G-SCF prophylaxis and antibiotic prophylaxis for FN was at the discretion of the treating physician in our study; however, 11 (22%) patients receiving primary or secondary prophylaxis with G-CSF showed a decreased rate of neutropenia. The use of primary prophylactic G-CSF to prevent FN may further reduce the toxicity, minimize the need for dose reduction, and improve patient compliance to chemotherapy.

A recent meta-analysis which examined trastuzumab-related cardiac toxicity in breast cancer patients showed an incidence of high-degree CHF of up to 4% [22]. Approximately 14% of patients who received adjuvant trastuzumab reportedly showed an asymptomatic decrease in LVEF [23]. In the present study, although 4% of patients showed a 20% decrease in LVEF, none of these patients manifested symptomatic CHF during treatment. However, it should be noted that only patients with no history of heart disease and those with normalized LVEF were enrolled in this study.

Although the treatment was delayed or discontinued in almost 30% of the patients due to severe AEs, the pCR rate of 57.3% upon per-protocol analysis in the patients who completed the full six courses was equivalent to the pCR rate of 53% on ITT analysis. We have no clear evidence to explain why this is. As per the results of a pooled analysis of 6377 patients with primary breast cancer receiving neoadjuvant chemotherapy (reported by von Minckwitz et al. [24]), the pCR rate for HER2-positive patients who did not receive trastuzumab was 23.3%, and that for those who received trastuzumab was 40.9%. We simply considered that the addition of trastuzumab to cytotoxic chemotherapy in patients with HER2-positive breast cancer would have the substantial benefit of increasing the pCR rate. Nevertheless, it is a noteworthy finding that the ORR of 90.8% in the per-protocol population was higher than the ORR (66%) in the ITT population. Ando et al. [21]. conducted a neoadjuvant study in which 181 Japanese patients with stage II/IIIA HER2-negative breast cancer were randomly assigned to four 3-week cycles of carboplatin (AUC of 5, day 1) in combination with weekly paclitaxel (80 mg/m^2^, days 1, 8, 15) followed by four 3-week cycles of cyclophosphamide, epirubicin, and 5-fluorouracil (CEF) or weekly paclitaxel followed by four 3-week cycles of CEF. Among the 88 patients treated with carboplatin and paclitaxel, a treatment delay or dose reduction of either paclitaxel or carboplatin was required in 27.3% of patients. Considering that there was no difference in pCR rate between the full-dose-treated patients and the dose-reduction/delayed patients, and the limited RDI with the current dosage of carboplatin with an AUC of 6, the TCbH regimen (which includes dose-reduced, e.g., AUC of 5) or delayed-schedule carboplatin may be more feasible for Japanese women.

The lack of a control arm and the small sample size are key limitations of our study. The required number of patients (*n* = 50) was initially determined for patients with HER2-positive breast cancer regardless of HR status. Therefore, the study design assessed that the treatment response would be insufficiently active when stratified by HR status.

In addition, evaluations of HER2 status (including IHC and FISH) were performed at different centers, which is a potential limitation. Centralized validation of the HER2 status is likely to yield more accurate selection of HER2-overexpressing breast cancer [25].

In conclusion, neoadjuvant chemotherapy with TCbH showed a high pCR rate in patients with HER2-positive breast cancer. Considering the high frequency of discontinuation/delay in chemotherapy due to AEs at late courses, the use of G-CSF supportive therapy is considered necessary, and dose reduction of carboplatin/docetaxel should be considered for some Japanese women. Neoadjuvant chemotherapy with TCbH could be a reasonable alternative for Japanese women in whom anthracycline is contraindicated.

## Electronic supplementary material

Below is the link to the electronic supplementary material.

Supplementary material 1 (PDF 23 kb)
