# Supplementary material for: Neoadjuvant chemotherapy with trastuzumab, docetaxel, and carboplatin administered every 3 weeks for Japanese women with HER2-positive primary breast cancer: efficacy and safety
Source: Int J Clin Oncol. 2017 May 25;22(5):880–6. doi: 10.1007/s10147-017-1136-8 (PMC5608788; doi:10.1007/s10147-017-1136-8)
Supplement: Supplementary file 1 — Supplementary material 1 (PDF 23 kb) [file 10147_2017_1136_MOESM1_ESM.pdf]

Supplemental Table.

| Parameter | Luminal/HER2 | | Non-luminal/HER2 | | |
|---|---|---|---|---|---|
| | n | (%) | n | (%) | *p* |
| Number | 25 | (100) | 25 | (100) | |
| Age, years | | | | | |
| Mean(SD) | 54.8(±10.0) | | 51.6(±9.8) | | 0.2 |
| Tumor, mm | | | | | |
| Mean(SD) | 39.5(±18.7) | | 32.4(±14.6) | | 0.1 |
| T-stage | | | | | |
| 1 | 3 | (12) | 1 | (4) | 0.2 |
| 2 | 17 | (68) | 16 | (64) | |
| 3 | 5 | (20) | 8 | (32) | |
| N-stage | | | | | |
| 0 | 8 | (32) | 9 | (36) | 0.9 |
| 1 | 17 | (68) | 16 | (64) | |

SD, standard deviation
